# Immunogenicity of the Oral Rabies Vaccine Strain SPBN GASGAS in Dogs Under Field Settings in Namibia

**DOI:** 10.3389/fvets.2021.737250

**Published:** 2021-10-25

**Authors:** Umberto Molini, Rainer Hassel, Steffen Ortmann, Ad Vos, Malaika Loschke, Albertina Shilongo, Conrad M. Freuling, Thomas Müller

**Affiliations:** ^1^School of Veterinary Medicine, University of Namibia, Windhoek, Namibia; ^2^Ceva Innovation Center, Ceva Santé Animale, Dessau-Roßlau, Germany; ^3^Directorate of Veterinary Services, Ministry of Agriculture, Water and Land Reform, Windhoek, Namibia; ^4^Institute of Molecular Virology and Cell Biology, Friedrich-Loeffler-Institut, WHO Collaborating Centre for Rabies Surveillance and Research, OIE Reference Laboratory for Rabies, Riems, Germany

**Keywords:** Africa, dogs, rabies, oral vaccination, SPBN GASGAS, neutralizing antibodies, binding antibodies

## Abstract

Dog-mediated rabies is endemic throughout Africa. While free-roaming dogs that play a crucial role in rabies transmission are often inaccessible for parenteral vaccination during mass dog vaccination campaigns, oral rabies vaccination (ORV) is considered to be a promising alternative to increase vaccination coverage in these hard-to-reach dogs. The acceptance of ORV as an efficient supplementary tool is still low, not least because of limited immunogenicity and field trial data in local dogs. In this study, the immunogenicity of the highly attenuated 3rd-generation oral rabies vaccine strain SPBN GASGAS in local free-roaming dogs from Namibia was assessed by determining the immune response in terms of seroconversion for up to 56 days post-vaccination. At two study sites, free-roaming dogs were vaccinated by administering the vaccine either by direct oral administration or *via* a vaccine-loaded egg bait. Pre- and post-vaccination blood samples were tested for rabies virus neutralizing as well as binding antibodies using standard serological assays. A multiple logistic regression (MLR) analysis was performed to determine a possible influence of study area, vaccination method, and vaccine dose on the seroconversion rate obtained. About 78% of the dogs vaccinated by the oral route seroconverted (enzyme-linked immunosorbent assay, ELISA), though the seroconversion as determined by a rapid fluorescence focus inhibition test (RFFIT) was much lower. None of the factors examined had a significant effect on the seroconversion rate. This study confirms the immunogenicity of the vaccine strain SPBN GASGAS and the potential utility of ORV for the control of dog-mediated rabies in African dogs.

## Introduction

Rabies is one of the zoonotic tropical diseases that remains neglected until today, with tens of thousands of humans still dying of rabies, mostly infected by rabid dogs (1). With highly efficacious rabies diagnostics as well as biologicals available, i.e., pre- and post-exposure prophylaxis, human rabies is clearly preventable. Also, broad-scale canine rabies elimination is both epidemiologically and operationally feasible and could be achieved across a wide range of settings in Africa and Asia (2). As a consequence, a global concept to eliminate dog-mediated human rabies by 2030 was initiated by the international rabies community and its stakeholders (3, 4). Using a true One Health concept, the Global Strategic Plan considers to control the disease at its animal source, the dog. It may appear straightforward to the outside world to vaccinate dogs. However, considering the differing socio-cultural and environmental conditions across canine rabies endemic regions in the world, it remains a challenge to achieve herd immunity in the population so that transmission stops. Mass dog vaccinations (MDV) using parenteral inactivated vaccines have been the primary means of rabies control in dogs. However, outside of North America (5, 6), Europe (7), and Latin America where this approach has been used successfully (7, 8), there are only a few examples of success for Asian countries including Japan, South Korea, Singapore, and Taiwan (9–12) where the same applies. In contrast, in Africa, sustained success of MDV using parenteral vaccines is still scarce.

The central problem in Asia and Africa are huge numbers of free-roaming dogs that are often inaccessible for parenteral vaccination during MDV campaigns (13). To increase vaccination coverage in these hard-to-reach dogs, the concept of oral rabies vaccination (ORV) of dogs as a complementary tool to mass parenteral dog vaccination has been proposed (14). Unfortunately, it is still the most underused of all tools in the fight against dog-mediated rabies (15), however, the concept has gained reviving interest in recent years (16–20). In fact, by targeting free-roaming and partly inaccessible dogs, ORV does not only increase the vaccination coverage of the overall dog population (16, 21) but also reaches those animals that are considered to play a key role in the transmission of rabies (16, 22, 23). While there are numerous oral rabies vaccines available (24), only vaccines with the highest safety profile that have been licensed for wildlife in accordance with international standards (25) prequalify for use in dogs. Next to safety assessments, immunogenicity studies and field trials are considered an essential part for either conditional or full-fledged licensure of oral rabies vaccines for dogs (15). However, there are still very limited experimental and field data for local free-roaming dogs available.

Therefore, the objective of this study was to assess the immunogenicity of SPBN GASGAS, a 3rd-generation vaccine licensed for wildlife (26, 27), in free-roaming dogs under African field settings. We aimed at determining the immune response post-vaccination after administration of the vaccine *via* an egg-flavored bait or direct oral administration. Also, we wanted to elucidate the impact of study area, vaccination method, and vaccine dose on the seroconversion obtained.

## Materials and Methods

### Study Design

The study sites were located in two different rural communities, Groot Aub and Ongombo West, about 56 km south and 42 km northeast of Windhoek, Namibia, respectively. Vaccinations were carried out in Groot Aub and Ongombo West at the end of February and end of September 2020, respectively. Owners were asked to bring their dogs to a central vaccination point established in both of the study sites for vaccination at a predetermined date that had been previously announced. All dogs were identified by morphological features, owner details, and two photographs and assigned a study number.

After the general health conditions were checked by a veterinarian, dogs were vaccinated with the oral rabies vaccine strain SPBN GASGAS (26–28) with a titer of 10∧7.5 or 10∧7.1 FFU/ml, using an egg-flavored bait with the vaccine filled in a soft blister (3.0 ml) (Tables 1, 2). The egg bait was essentially the same as used in field studies in local dogs from other areas (29–31) or large-scale oral rabies field trials in dogs from Thailand (32). For comparison, a few dogs were vaccinated by direct oral application (d.o.a., 3.0 ml), when they refused to consume a bait, or parenterally using a commercial inactivated vaccine (RABISIN® ad us. vet., Boehringer Ingelheim, Germany), with the latter serving as positive controls. Bait uptake and blister perforation were closely monitored.

**Table 1:** Seroconversion of local Namibian dogs after oral (bait and d.o.a.) and parenteral vaccination according to RFFIT (≥0.5 IU/ml) and ELISA (≥40% PB).

**Route of administration**	**rVNA (RFFIT)**			**rVBA (ELISA)**		
	***n***	***N***	**%**	***n***	***N***	**%**
Oral	18	34	52.94	26	33	78.79
Bait	13	26	50.00	20	26	76.92
d.o.a.	5	8	62.50	6	7	85.71
Parenteral	2	2	100.00	2	2	100.00

*Only animals that tested seronegative prior to vaccination are included (n, number of animals that tested seropositive; N, number of animals tested; ELISA, enzyme-linked immunosorbent assay; rVBA, rabies-specific binding antibodies; RFFIT, rapid fluorescence focus inhibition test; rVNA, rabies virus-neutralizing antibody; d.o.a., direct oral administration)*.

**Table 2:** Parameter estimates from the multiple logistic regression model (linear without interactions) indicating factors associated with seroconversion (ELISA and RFFIT) in local dogs in Namibia.

	**rVNA (RFFIT)**			**rVBA (ELISA)**		
	**Coefficient**	**Odds ratio**	**95% CI**	**Coefficient**	**Odds ratio**	**95% CI**
**Factors**						
Intercept	1.216	3.373	1.029–15.100	1.699	5.466	1.455–35.500
**Study area**						
Ongombo West	−2.850	0.058	0.006–0.347	−1.119	0.327	0.037–2.150
**Route**						
d.o.a.	1.115	3.175	0.456–31.570	0.856	2.353	0.285–51.840
**Dose**												
High	1.453	4.274	0.500–47.020	0.919	2.506	0.259–57.940

*Groot Aub, bait, and low dose were set as reference in MLR; 95% CI, 95% confidence interval of odds ratio*.

Blood samples were collected -14 (B0) days pre-vaccination and 28 (B1) days post-vaccination (dpv), while at the time point of vaccination (V), no blood samples were taken. The dogs were manually restrained for blood collection from the cephalic or the jugular vein. Subsequently, blood samples were labeled with the dogs' study number and placed in a cooling box for transportation. After arrival in the laboratory, all blood samples were centrifuged at 1,000 *g* for 10 min within 24 h of collection, aliquoted, and the serum stored at −20°C until serological testing.

In order to obtain an acceptable sample size per study area, dog owners in Ongombo West willing to participate with their dogs in the study were paid an incentive for following the study protocol. Because of unforeseen circumstances due to a sudden implementation of COVID-19 restrictions, blood sampling at 28 dpv was impaired, so study sites were revisited 56 dpv in an attempt to obtain a complete set of samples from all vaccinated dogs.

### Diagnostic Assays

Prior to testing, sera were inactivated at 56°C for 30 min. Sera were tested for rabies-specific binding antibodies (rVBA) in a commercial blocking enzyme-linked immunosorbent assay (ELISA) (BioPro Rabies ELISA, Czech Republic) essentially as described (33, 34) by using positive (PC) and negative controls (NC) provided by the manufacturer and following validity parameters and characteristics as stated in the kit insert.

Additionally, sera were tested for the presence of rabies virus-neutralizing antibodies (rVNAs) in a modified rapid fluorescence focus inhibition test (RFFIT) using RABV (CVS-11) as test virus and BHK21-BSR/5 (Collection of Cell Lines in Veterinary Medicine—catalog N° CCLV-RIE 0194/260) cells with VNA titers expressed in international units per milliliter (IU/ml) as described (35). The calibrated WHO international standard immunoglobulin (2nd human rabies immunoglobulin preparation, National Institute for Standards and Control, Potters Bar, UK) adjusted to 0.5 IU and a naive dog serum served as PC and NC, respectively.

Sera were considered seropositive for rVBA if sera showed a percentage of inhibition compared to the negative serum >40% in ELISA or for rVNAs if titers were ≥0.5 IU/ml (25, 36).

### Statistical Analysis

A multiple logistic regression (MLR) analysis was performed for seroconversion in the orally vaccinated dogs. It was determined if the study area (Groot Aub or Ongombo West), vaccination method (bait or d.o.a.), and vaccine dose (low or high) had an influence on seroconversion as determined by ELISA and RFFIT. For the MLR analysis, GraphPad Prism 9.0 (GraphPad Software Inc., San Diego, CA, USA) was used.

### Ethical Considerations

This preliminary study was part of a larger approved project on oral vaccination of dogs in Namibia sponsored by the German Ministry of Health under the Global Health Protection Programs (Project number Ri-0755) and implemented as an agreed activity in the frame of the Official National Dog Rabies Control Program issued by the Namibian Directorate of Veterinary Services (DVS) at the Ministry of Agriculture, Water, Forestry and Land Reform (MoA). In this respect, the study was regarded a disease control trial, and ethical committee approval was not warranted. The study was approved by the appropriate authority within the MoA, Namibia. Manipulations such as vaccination and blood sampling of dogs were only conducted after elaborate explanatory meetings and under the premise that the dog owner had previously given his/her written consent. Institutional biosecurity and safety procedures for handling vaccines, samples, reagents, and virus were followed.

## Results

A total of 85 local Namibian dogs were initially included and bled (B0) from both study sites; Groot Aub (*N* = 51) and Ongombo West (*N* = 34). However, on the days of vaccination, 28 dogs were not brought to the vaccination sites. Hence, a total of 57 dogs were vaccinated; 36 dogs were offered a bait, 12 dogs received the oral rabies vaccine by d.o.a., and 9 dogs were vaccinated by the parenteral route. For 10 dogs (27.78%) offered a bait, an impaired uptake was observed. These dogs either initially refused to take a bait and it needed two or three attempts before they accepted it or hastily consumed the bait offered before swallowing the sachet during bait consumption; the other 26 dogs discarded the perforated sachet.

Unfortunately, from 17 (29.82%) of the 57 treated dogs, no blood sample could be collected after vaccination. Of the 40 remaining dogs, four dogs each tested positive for rVBA and rVNA prior to vaccination (B0) and were omitted from statistical analysis. Furthermore, no blood sample was collected from three of 36 remaining dogs on 28 dpv, and therefore, the results of the blood sample collected on 56 dpv from these three dogs were used.

**Figure 1:**
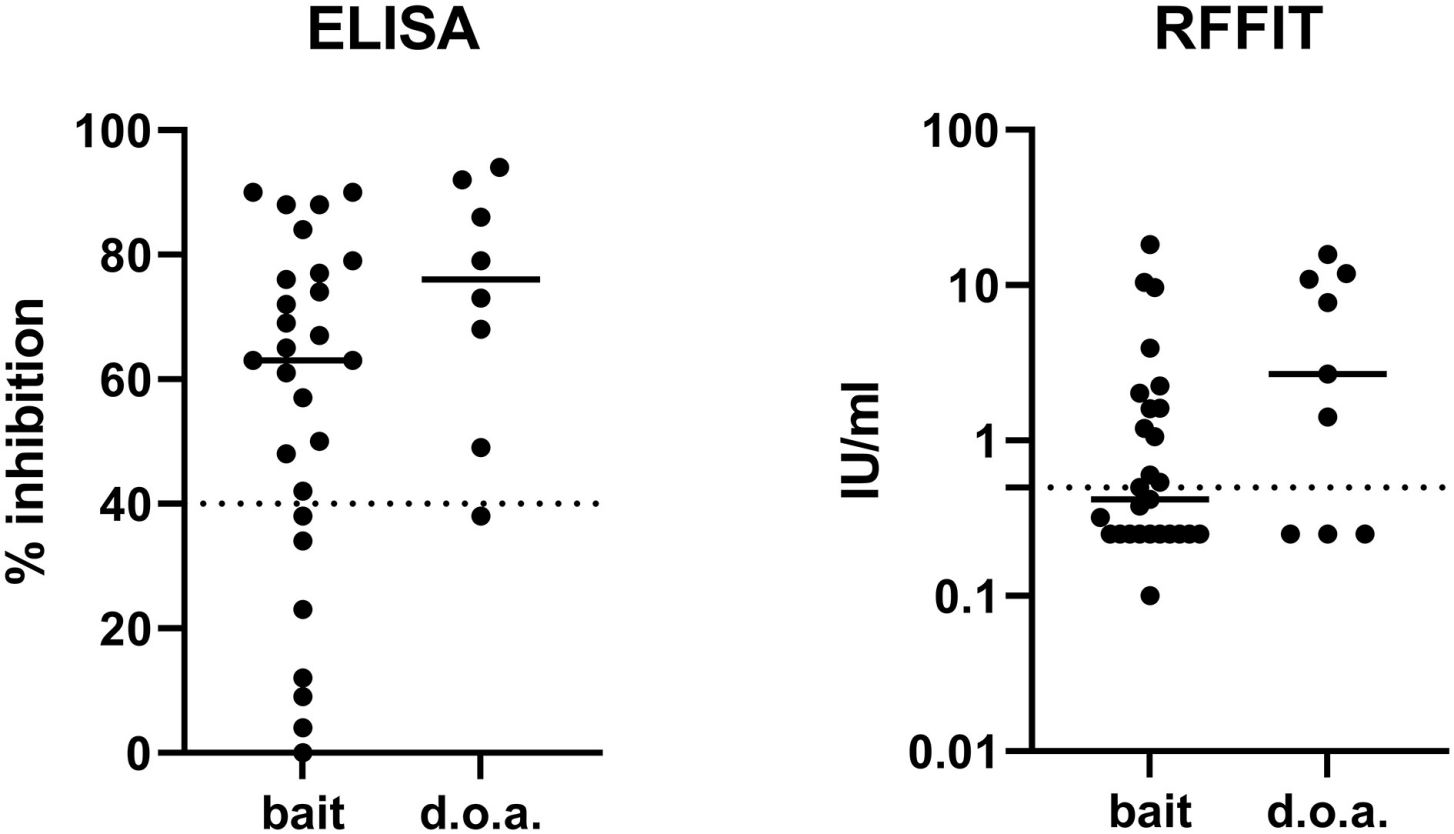
The immune response measured by ELISA (rVBA) and RFFIT (rVNA) after oral bait or direct oral administration (d.o.a.). Of several samples that tested negative in RFFIT, no exact value could be estimated (<0.5 IU/ml); thus, an average value of 0.25 IU/ml (range 0–0.5 IU/ml) was set for these samples. The solid lines indicate the means of the measured values in ELISA and RFFIT, while the cut-off levels for both serological tests are indicated by the dashed lines. ELISA, enzyme-linked immunosorbent assay; rVBA, rabies-specific binding antibodies; RFFIT, rapid fluorescence focus inhibition test; rVNA, rabies virus-neutralizing antibody.

The seroconversion rate of the dogs vaccinated is shown in Table 1. Almost 80% of the dogs vaccinated by the oral route seroconverted (ELISA), and the seroconversion rate using the 0.5 IU/ml cut-off for the RFFIT was considerably lower at 53% (Figure 1). Six of the eight dogs that swallowed the sachet during bait consumption after offering them a bait and were bled at 28 dpv showed rVBA in ELISA, while only two had rVNA titers ≥0.5 IU/ml. As we only have two data sets for dogs vaccinated by the parenteral route, they are not included in the statistical analysis; both dogs seroconverted.

The results of the MLR showed that none of the factors examined (study area, route, and dose) had a significant effect on seroconversion as determined in ELISA (Table 2). However, for rVNAs as measured by RFFIT, the seroconversion rate was much lower in Ongombo West than in Groot Aub. Only 31.58% of the dogs seroconverted (≥0.5 IU/ml) in Ongombo West, compared to 80% in Groot Aub (Table 3).

**Table 3:** Seroconversion rate of dogs after oral [bait and direct oral administration (d.o.a.)] vaccination according to RFFIT (≥0.5 IU/ml) and ELISA (≥40% PB) according to study site.

**Study area**	**rVNA (RFFIT)**			**rVBA (ELISA)**		
	***n***	***N***	**%**	***n***	***N***	**%**
Groot Aub	12	15	80.00	12	14	85.71
Ongombo West	6	19	31.58	14	19	73.68

*Only animals that tested seronegative prior to vaccination are included (n, number of animals that tested seropositive; N, number of animals tested)*.

## Discussion

This is the first study assessing the immune response of the 3rd-generation oral rabies vaccine strain SPBN GASGAS in local, free-roaming dogs under Southern African conditions. In settings where traditional parenteral methods have failed to reach adequate vaccination coverages against rabies, oral rabies vaccines can be very helpful in reaching adequate herd immunity (15), particularly in free-roaming dogs, which are considered to play a key role in the transmission of rabies (16, 22, 23). In great parts of Africa, actually, most free-roaming dogs are owned and are deemed accessible for parenteral vaccination at MDV campaigns (37–39). However, experience shows that many of these MDV campaigns are not able to reach a sufficient number of dogs (40).

Several studies examined the immunogenicity and/or efficacy of oral rabies vaccines in dogs. Most of these studies were conducted under (semi-) controlled laboratory conditions using laboratory dogs or local dogs adapted to dog kennel conditions (41–48).

Generally, the immune response in the target population of free-roaming dogs under field conditions has rarely been investigated both after vaccination with inactivated rabies vaccines (49, 50) and oral rabies vaccines (51). Often, these animals are in poor condition compared to dogs well-looked after. Free-roaming dogs are generally malnourished as their diet is of low quality and/or quantity, having a negative impact on immunity (52, 53). Also, these animals are usually infected by endo- and ectoparasites or other immunosuppressive infectious agents (54). For example, canine distemper virus (CDV) can infect and modulate the antigen presenting properties of dendritic cells and thus influence the immune response against CDV and other concomitant infections/vaccinations (55). Hence, the possibility of immunosuppression through various stressors may reduce seroconversion in subpopulations of free-roaming dogs after rabies vaccination and the duration of immunity of seroconverted dogs and result in a decline in the vaccination coverage (50). Consequently, investigating the effectiveness of oral vaccination in free-roaming dogs under field conditions prior to large-scale implementation is critical.

Unfortunately, the sample size particularly in the first study area of Groot Aub continuously decreased during the course of the project. Initially, it was planned to obtain blood samples after 4 weeks, as day 28 post-vaccination seroconversion appears to be a good predictor for protection (35). However, due to the emerging SARS-CoV-2 pandemic in February 2020 and associated restrictions, subsequent sampling in Groot Aub was delayed and compliance was heavily reduced. To cope with the situation, it was decided to return to the study sites 56 dpv. However, only a few more blood samples could be collected and included in the analysis. In order not to jeopardize the outcome of the study by a similar situation occurring in Ongombo West half a year later, an incentive to the owners was provided. This ensured full compliance with study protocol.

Nevertheless, independent of study area, route, and dose, 78.8% of the free-roaming Namibian dogs vaccinated orally (either offered a bait or d.o.a.) developed a detectable immune response post-vaccination as measured by ELISA (rVBA), while the seroconversion rate in terms of rVNAs as measured by RFFIT was lower (Tables 1, 3). The discrepancy is due to the fact that methods for measuring rabies immunity vary with regard to the humoral component measured (i.e., the Ig subclass or functional activity) and performance characteristics (i.e., specificity or sensitivity). When results of testing sera by both RFFIT and ELISA kit are compared for a particular target species, similar measures of sensitivity and specificity are obtained only when using different cut-off values for both assays (56). However, in our study, we followed international standards and recommendations of the manufacturer regarding cut-off values. As to why only 31.58% of the dogs in Ongombo West compared to 80% in Groot Aub had positive rVNA remains elusive (Table 3). However, while the level of 0.5 IU/ml was established as an indication of adequate vaccination rather than protection in humans, when the RFFIT is employed for other species (not humans), the accepted level of 0.5 IU/ml may not apply (56). So, the seroconversion rate based on rVNAs might even be higher; however, if the cut-off level is set too low, it gets more difficult to distinguish specific from non-specific neutralizing activity of a test serum. In any case, data from experimental studies clearly showed that rVBAs are a better predictor for protection than rVNAs (35).

The seroconversion rate obtained in our study is consistent to those found in local dogs vaccinated by the oral route under field settings in Haiti using the same SPBN GASGAS vaccine and ELISA (51). In a recent immunogenicity study with local Thai dogs kept in a dog shelter, 100% of the dogs vaccinated by the oral route had detectable levels of antibodies 28 dpv, again using the same vaccine and ELISA. These dogs in Thailand, however, had received vaccination against some common infectious diseases (canine distemper, parvovirus infection, adenovirus infection, bronchitis, and leptospirosis). They also received helminthic treatment when they were around 3 months old (48). Finally, the animals were fed on a daily basis, receiving commercially available high-quality pet food, hence superior conditions compared to the dogs used in the Namibian and the Haitian study.

However, the difference in seroconversion after oral vaccination with SPBN GASGAS between the study in Thai shelter dogs and the two studies in free-roaming dogs from Haiti and Namibia most likely cannot only be explained just by the physical condition of the dogs involved. It has been shown, for example, that the development of antibodies is slower after oral vaccination compared to parenteral vaccination (48). In the Thai dog study, the highest levels of antibodies were not reached before 4 weeks post-vaccination in dogs orally vaccinated with SPBN GASGAS (48). Therefore, the short interval between vaccination and sampling (17–20 dpv) may explain the relatively low seroconversion rate for the Haiti study (51). Another factor that most likely affected the seroconversion rate in the latter study was the study design; the baseline blood sample was collected immediately after bait consumption (51). Hence, the free-roaming dogs were surrounded by the vaccination team to prevent it from wandering off after bait consumption. This made the dogs anxious, negatively influencing bait handling and consumption and, consequently, vaccine virus release in the oral cavity. If the vaccine bait is swallowed completely without chewing, the vaccine is not discharged in the oral cavity, or if such a cornered animal chews half-hearted on the bait, most vaccine is often spilled on the floor instead of being absorbed in the oral cavity.

Essential for a successful vaccination attempt is therefore not only an efficacious vaccine and attractive bait but also the circumstances under which baits are offered to the dogs. In this respect, a likely stressful situation occurred in the present study in Namibia. Although blood samples were collected several weeks prior to vaccination, for logistical reasons, most animals were not offered a bait on their own premises as would be done under normal circumstances. Instead, the normally free-roaming dogs were brought to a central vaccination point in the study area where they were offered a bait. Hence, differing environmental conditions may have had a negative effect on bait consumption (handling) of individual dogs. Obviously, many of them were stressed due to the fact that they were on a leash to which they are usually not accustomed with many other strange dogs and humans around them in unfamiliar territory. Although the bait acceptance rate equaled those of other studies with the same bait (29–31), bait uptake and handling were impaired during the present study, as vaccine baits were often initially refused or hastily consumed as documented for at least 10 dogs. These animals hardly chewed on the baits before swallowing it completely, including the most likely unperforated sachet. Hence, under such suboptimal conditions, it can be expected that in some dogs, an inadequate immune response after bait consumption was induced, as the vaccine was not sufficiently or not released at all in the oral cavity. This might help to explain the lower seroconversion obtained in RFFIT as six of eight of these dogs sampled 28 dpv seroconverted in ELISA, while only two showed rVNA in RFFIT. However, a recent field study in India and Thailand showed that when free-roaming dogs are offered a bait directly when encountered in their familiar environment, most animals will accept the bait readily and perforate the sachet (31, 32). Hence, it can be assumed that under real-scenario conditions, bait uptake most likely will not be impacted, resulting in an even higher seroconversion rate post-vaccination. Also, when data sets of rabies-binding and -neutralizing antibodies from animals experimentally immunized with both live attenuated or recombinant oral rabies vaccines were examined, an analysis suggested that, though rVNA are expected to reflect *in vivo* protection, rVBA (ELISA) obtained at 28 dpv were a better predictor of protection against lethal rabies infection (35).

## Conclusions

The results demonstrated that the great majority of orally vaccinated free-roaming Namibian dogs mount an immune response. The seroconversion rate in this study was slightly lower as compared to other immunogenicity studies under field and laboratory conditions, which we speculate is most likely associated with the central-point-vaccination (CPV) protocol used. Nevertheless, the study confirmed that local African dogs like any other local dogs across the world are very likely to develop an adequate immune response after oral vaccination with the 3rd-generation oral rabies vaccine strain SPBN GASGAS and are protected against rabies. These results underline the potential of ORV as an important tool for targeting hard-to-reach free-roaming dogs in mass dog vaccination campaigns under African settings. Rapid implementation of field trials is needed to see how ORV can be effectively and cost-efficiently integrated into African mass dog vaccination strategies at large scale.

## Data Availability Statement

The original contributions presented in the study are included in the article/supplementary material, further inquiries can be directed to the corresponding author.

## Ethics Statement

Ethical review and approval was not required for the animal study because this preliminary study was part of a larger approved project on oral vaccination of dogs in Namibia sponsored by the German Ministry of Health under the Global Health Protection Programs (Project number Ri-0755) and implemented as an agreed activity in the frame of the Official National Dog Rabies Control Program issued by the Namibian Directorate of Veterinary Services (DVS) at the Ministry of Agriculture, Water, Forestry and Land Use (MoA). In this respect, the study was regarded a disease control trial and Ethical Committee approval was not warranted. The study was approved by the appropriate authority within the MoA, Namibia. Manipulations such as vaccination and blood sampling of dogs were only conducted after elaborate explanatory meetings and under the premise that the dog owner had previously given his/her written consent. Written informed consent was obtained from the owners for the participation of their animals in this study.

## Author Contributions

UM, RH, ML, and SO planned and conducted the vaccination campaigns in the two rural communities, collected blood samples, and were responsible for data curation. AS was responsible for resource allocation and overall supervision of the study. SO developed and produced the egg-flavored baits. TM, UM, and CF were responsible for serological testing of sera, analyzed data, wrote, reviewed, and edited the manuscript. AV conceived the ideas, conducted the statistical analysis, and contributed to writing and reviewing of the manuscript. All authors contributed to the article and approved the submitted version.

## Funding

Members of the FLI were supported by the German Ministry of Health through grant Ri-0755.

## Conflict of Interest

AV and SO are employees of the Ceva Innovation Center GmbH, Dessau, Germany. This company is manufacturing oral rabies vaccine baits for wildlife. The remaining authors declare that the research was conducted in the absence of any commercial or financial relationships that could be construed as a potential conflict of interest. The collection, analyses, and interpretation of data, the drafting of the manuscript, and the subsequent decision to publish was jointly made by all co-authors. The reviewer SM declared a past co-authorship with several of the authors TM and CF, to the handling editor.

## Publisher's Note

All claims expressed in this article are solely those of the authors and do not necessarily represent those of their affiliated organizations, or those of the publisher, the editors and the reviewers. Any product that may be evaluated in this article, or claim that may be made by its manufacturer, is not guaranteed or endorsed by the publisher.
